# Severe Delayed Hypersensitivity Reaction at Guselkumab Injection Site

**DOI:** 10.1111/cod.70034

**Published:** 2025-10-30

**Authors:** Salomé Allichon, Julie Litovsky, Michèle Sanchez, Céline Girard, Guillaume Granier, Luc Durand, Maxime Henrion, Christelle Philibert, Cosette Le Souder, Nadia Raison‐Peyron

**Affiliations:** ^1^ Department of Dermatology Montpellier University Hospital Montpellier France; ^2^ Department of Dermatology Hospital of Avignon Avignon France; ^3^ Department of Pathology Hospital of Avignon Avignon France; ^4^ Department of Pathology Montpellier University Hospital Montpellier France; ^5^ Department of Pharmacovigilance Montpellier University Hospital Montpellier France

**Keywords:** adverse reaction, anti‐IL23, biologic agents, case report, delayed hypersensitivity, guselkumab, injection site reaction, psoriasis

Injection site reactions (ISRs) due to biologics are frequent with an incidence ranging from 0.5% to 40%, depending on the medication. However, the origin of these reactions often remains unclear [1, 2]. We present a case of hypersensitivity reaction at the guselkumab injection site.

## 1 Case Report

A 62‐year‐old woman presented with severe plaque psoriasis and psoriatic arthritis, with a history of allergic asthma and contact dermatitis to fragrances. Plaque psoriasis started at age 8 and arthritis symptoms appeared around age 50, worsening at age 56. She was previously treated with methotrexate, which was stopped because of hepatic cytolysis. Subsequently, treatment with subcutaneous guselkumab (Tremfya, Janssen‐Cilag) was initiated at 100 mg at Week 0, Week 4, and then administered every 8 weeks in the thigh.

**FIGURE 1:**
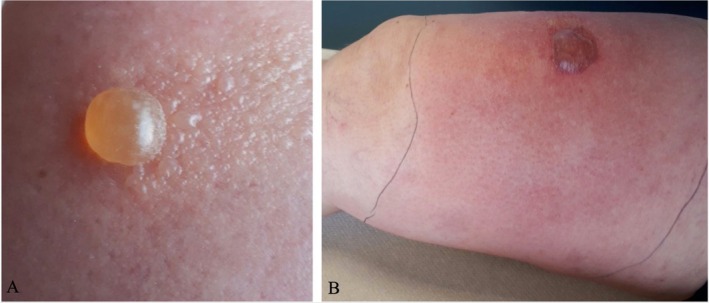
(A, B) Injection site reaction after the third injection of guselkumab, (A) 24 h after, (B) 48 h after.

Two days after the first injection, she developed a large itchy erythematous and edematous plaque at the site of injection. After the second injection, 4 weeks later, the same reaction occurred but more severe within 24 h. Following the third injection, 8 weeks later, a large edematous plaque with vesicles and a bulla secondarily hemorrhagic appeared within one hour (Figure 1A,B). Blood tests showed a normal eosinophil count and a C reactive protein of 18.6 mg/L. No skin biopsy was performed. The last episode resolved within 10 days after treatment with antihistamines and topical corticosteroid. An antiseptic solution Biseptine and a bandage Mepilex border flex were also used at the time of the third injection but were later used without further reaction.

Patch tests performed with the European baseline series and polysorbate 80 (5% pet), an excipient in Tremfya (Chemotechnique Diagnostics, Vellinge, Sweden) and personal products such as Biseptine, Tremfya and Mepilex border flex, all tested ‘as is’ were all negative on Day 2 and Day 4 except for fragrance mix I with a past relevance. An intradermal test (IDT) done with 0.03 mL of Tremfya (100 mg/mL) showed an immediate positive reaction with an erythematous papule and extensive erythema after 30 min. Delayed reading, at 24 h, revealed persistent inflammation with a central vesicle. Biopsy demonstrated perivascular and interstitial inflammation, with a dermal eosinophilic infiltrate and flame figures suggesting (but not specific) of eosinophilic cellulitis (Figure S1). Direct immunofluorescence showed isolated C3 vascular deposition without specificity. Hypersensitivity to polysorbate 80 was excluded by a negative IDT to triamcinolone acetonide (Kenacort retard, 40 mg/mL), which also contains polysorbate 80 [3]. The IDT with Tremfya in a patient who received this treatment without adverse reaction was negative at both readings.

One year later, the patient received the etanercept biosimilar (Benepali, Samsung Bioepis, Netherlands), a fusion protein targeting TNF alpha and free of polysorbate. After the first subcutaneous injection, she developed an ISR with a large erythematous plaque with no vesicle or bullae within 24 h, lasting 4 days. Skin biopsy findings were compatible with urticaria. Injection of the same volume of physiological serum subcutaneously elicited no reaction, ruling out a pathergy process. A decrease in intensity of the local reactions after each injection (4) lead to the conclusion of common reactions at the injection site. However, the treatment was discontinued due to inefficacity. She is currently being treated with secukinumab, a human anti‐interleukin‐17A monoclonal antibody (Cosentyx, Rueil‐Malmaison, Novartis Pharma) which contains polysorbate 80 with excellent tolerance and efficacy.

## 2 Discussion

ISRs related to biologics are increasingly reported as they are more commonly used in inflammatory diseases. These reactions are usually mild and rarely require treatment discontinuation. Most ISRs are irritative and due to inappropriate injection technique [4].

Complete explorations to elucidate the underlying mechanism of ISRs—whether irritative or allergic—and to identify the culprit (either the active ingredient or an excipient) through in vivo tests, in vitro assays, or drug‐provocation tests are not routinely performed, and only a few well‐documented case reports have been reported [5, 6].

Guselkumab is a human immunoglobulin G monoclonal antibody targeting IL‐23 developed for the treatment of psoriatic arthritis and moderate‐to‐severe plaque psoriasis [7]. It is administered subcutaneously. ISRs such as erythema, hematoma, edema, pruritus, pain, induration, rash and urticaria have been frequently described (1.1% with Tremfya 100 mg, 8.9% with Tremfya 200 mg) and anaphylaxis has also been mentioned [8]. A case of nummular dermatitis has been reported after 3 months of guselkumab therapy in a 40‐year‐old man with a chronic history of refractory palmoplantar psoriasis [9].

ISRs diagnosed as eosinophil cellulitis have been reported with multiple biologics, in some instances involving lesions distant from the injection site [10, 11].

In our case, the increasing intensity and rapid onset of the inflammatory reaction with successive injections suggest an immune‐induced mechanism.

In conclusion, this first reported case of hypersensitivity to guselkumab highlights the need for heightened vigilance regarding biological medicines and the current lack of knowledge on potential cross‐reactivity among monoclonal antibodies.

## Consent

The patient in this manuscript has given written informed consent to publication of their case details and photographs.

## Conflicts of Interest

Dr. Michèle Sanchez reports grants/contracts from Sanofi, Janssen, Almirall, Boehringer, Novartis, Abbvie. The other authors declare no conflicts of interest.

## Supporting information


**Figure S1:** Anatomopathological analysis of a skin biopsy 24 h after the intradermal test to guselkumab. Haematoxylin Eosin stain. (A) (magnification ×100) slightly hyperplastic epidermis with orthokeratosis, associated with an inflammatory infiltrate involving the entire height of the dermis of perivascular (*) and interstitial (arrow) topography. (B) (magnification ×200) the inflammatory infiltrate is mainly composed of eosinophilic polynuclears, some of which are degranulated. This inflammatory infiltrate is accompanied by ‘flaming’ (*) alterations to the collagen fibres (resulting from the deposition of eosinophilic basic protein on the collagen fibres). There are no obvious signs of vasculitis.

## Data Availability

The data that support the findings of this study are available from the corresponding author upon reasonable request.
