# Supplementary figures and images for: Severe Delayed Hypersensitivity Reaction at Guselkumab Injection Site
Source: Contact Dermatitis. 2025 Oct 30;94(1):89–91. doi: 10.1111/cod.70034 (PMC12695513; doi:10.1111/cod.70034)

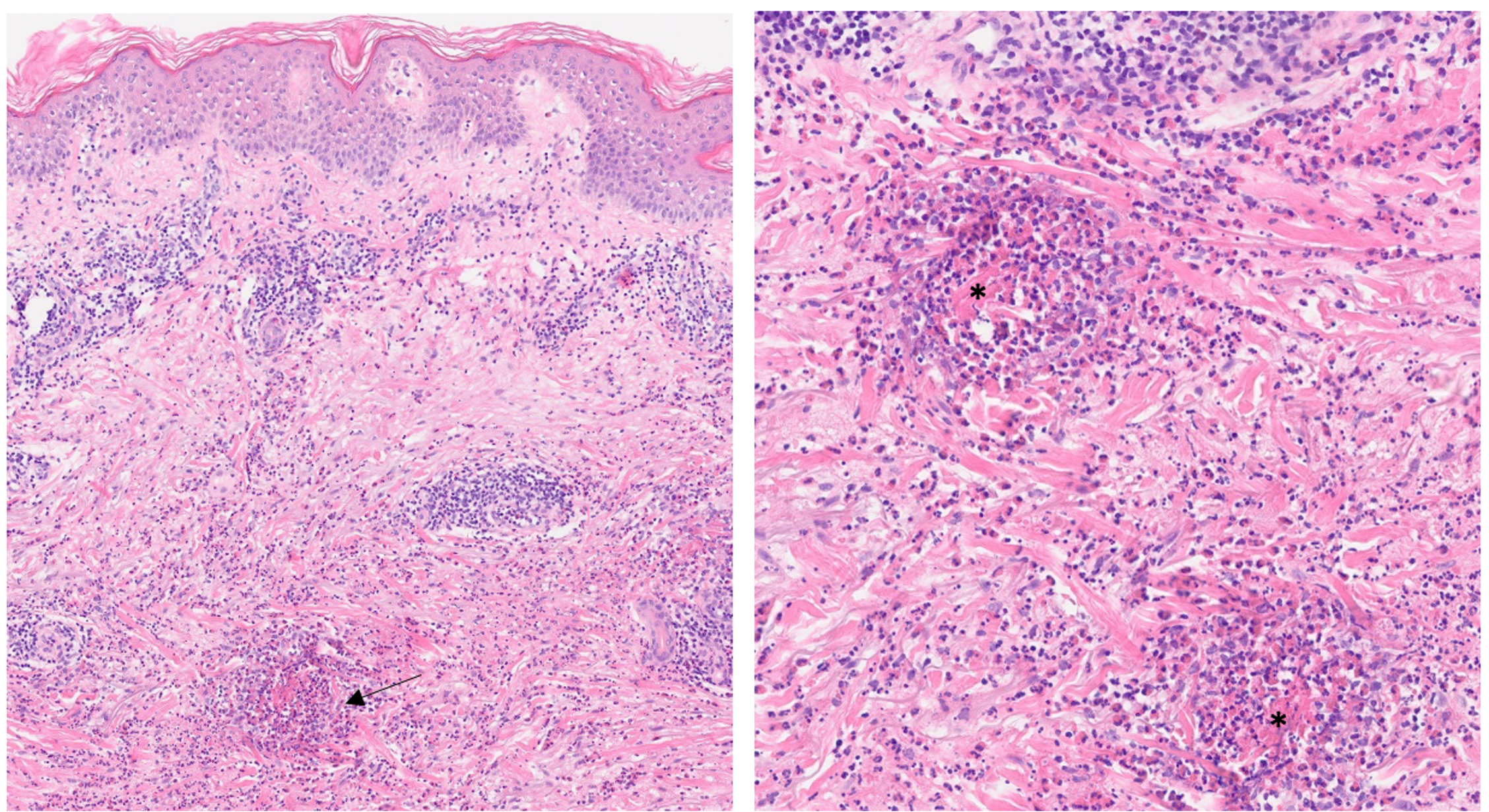

Supplement: Supplementary file 1 — Figure S1: Anatomopathological analysis of a skin biopsy 24 h after the intradermal test to guselkumab. Haematoxylin Eosin stain. (A) (magnification ×100) slightly hyperplastic epidermis with orthokeratosis, associated with an inflammatory infiltrate involving the entire height of the dermis of perivascular (*) and interstitial (arrow) topography. (B) (magnification ×200) the inflammatory infiltrate is mainly composed of eosinophilic polynuclears, some of which are degranulated. This inflammatory infiltrate is accompanied by ‘flaming’ (*) alterations to the collagen fibres (resulting from the deposition of eosinophilic basic protein on the collagen fibres). There are no obvious signs of vasculitis. [file COD-94-89-s001.png]
